# Electronic Structure Evolution with Composition Alteration of Rh_*x*_Cu_*y*_ Alloy Nanoparticles

**DOI:** 10.1038/srep41264

**Published:** 2017-01-25

**Authors:** Natalia Palina, Osami Sakata, L. S. R. Kumara, Chulho Song, Katsutoshi Sato, Katsutoshi Nagaoka, Tokutaro Komatsu, Hirokazu Kobayashi, Kohei Kusada, Hiroshi Kitagawa

**Affiliations:** 1 Synchrotron X-ray Station at SPring-8, Research Network and Facility Services Division, National Institute for Materials Science (NIMS), 1-1-1 Kouto, Sayo-cho, Sayo-gun, Hyogo 679-5148, Japan; 2 Synchrotron X-ray Group, Research Center for Advanced Measurement and Characterization, NIMS, 1-1-1 Kouto, Sayo-cho, Sayo-gun, Hyogo 679-5148, Japan; 3 Department of Materials Science and Engineering, School of Materials and Chemical Technology, Tokyo Institute of Technology, 4259-J3-16, Nagatsuta, Midori, Yokohama 226-8502, Japan; 4 Elements Strategy Initiative for Catalysts and Batteries, Kyoto University, 1-30 Goryo-Ohara, Nishikyo-ku, Kyoto 615-8245, Japan; 5 Department of Applied Chemistry, Faculty of Engineering, Oita University, 700 Dannoharu, Oita 870-1192, Japan; 6 School of Medicine, Nihon University, 30-1, Oyaguchi Kami-cho, Itabashi-ku, Tokyo 173-8610, Japan; 7 Division of Chemistry, Graduate School of Science, Kyoto University, Kitashirakawa Oiwake-cho, Sakyo-ku, Kyoto 606-8502, Japan; 8 Institute for Integrated Cell-Material Sciences (iCeMS), Kyoto University, Yoshida, Sakyo-ku, Kyoto 606-8501, Japan; 9 INAMORI Frontier Research Center, Kyushu University, 744 Motooka, Nishi-ku, Fukuoka 819-0395, Japan

## Abstract

The change in electronic structure of extremely small Rh_*x*_Cu_*y*_ alloy nanoparticles (NPs) with composition variation was investigated by core-level (CL) and valence-band (VB) hard X-ray photoelectron spectroscopy. A combination of CL and VB spectra analyses confirmed that intermetallic charge transfer occurs between Rh and Cu. This is an important compensation mechanism that helps to explain the relationship between the catalytic activity and composition of Rh_*x*_Cu_*y*_ alloy NPs. For monometallic Rh and Rh-rich alloy (Rh_0.77_Cu_0.23_) NPs, the formation of Rh surface oxide with a non-integer oxidation state (Rh^(3−δ)+^) resulted in high catalytic activity. Conversely, for alloy NPs with comparable Rh:Cu ratio (Rh_0.53_Cu_0.47_ and Rh_0.50_Cu_0.50_), the decreased fraction of catalytically active Rh^(3−δ)+^ oxide is compensated by charge transfer from Cu to Rh. As a result, ensuring negligible change in the catalytic activities of the NPs with comparable Rh:Cu ratio to those of Rh-rich and monometallic Rh NPs.

Interest in alloy catalysts is driven by initial industry demand for bimetallic catalysts^1^,^2^,^3^ and quantum theory of alloys; in particular, theories predicting the surface composition of alloys^4^,^5^. Compared with their monometallic components, bimetallic systems are also expected to display unique properties that are not characteristic of the monometallic constituents because of a possible synergistic effect between the two metals. Most catalytically active metals are transition metals with frontier d-orbitals, so considerable effort has been dedicated to d-band modification. Hammer and Nørskov developed the d-band center model, which correlates electronic structure to catalyst reactivity^6^,^7^. This model introduces the following ideas: alloying causes (i) narrowing of the d-band, which is the main change, and either (ii) electron transfer to one alloy component or (iii) certain rehybridisation of all alloy components. The d-band center model readily explains some experimental data^8^,^9^,^10^,^11^. It is believed that the narrowing of d-band width is a general trend in alloys. To avoid confusion, we should stress that changes in electronic structure that are caused by post-fabrication modifications such as exposure to reactive gases, annealing or other intentional surface modification can possibly lead to broadening of the d-band width and are not considered here.

With the rapid growth of the number of automobiles in the modern world, effective three-way catalysts (TWCs) to purify harmful exhaust gases including nitrogen oxides (NOx), carbon monoxide (CO) and hydrocarbons have increasingly been viewed as a necessary technology to prevent serious atmospheric pollution^12^,^13^,^14^,^15^. As their name reflects, TWCs are used for the simultaneous and effective treatment of the major pollutants stimulated by catalysts. When the reaction conditions are nearing theoretical air-to-fuel ratio, harmful pollutants can be effectively converted to their harmless stable products: carbon dioxide (CO_2_), water (H_2_O) and nitrogen (N_2_). TWCs typically consist of (i) a stainless steel-based monolithic substrate with a honeycomb structure, (ii) a support with high surface area and oxygen storage materials coated on the monolith, (iii) noble metals (Pt, Pd and Rh) as the active catalysts, and (iv) metal oxides that mainly function as promoters^16^,^17^. Rhodium (Rh) is an indispensable component of TWCs because of its high catalytic activity for NOx reduction^18^ and partial oxidation of higher hydrocarbons^19^. However, Rh is one of the most expensive metals because of its scarcity. Therefore, much research has focused on removing the need for excessive use of Rh. This has triggered efforts to synthesise cost-effective, highly stable materials that exhibit catalytic activity comparable with that of noble metals. Alloys of Rh with Cu are candidates for effective TWCs based on the concepts of density of states (DOS) engineering and non-equilibrium synthesis^20^,^21^,^22^.

To identify alloy compositions suitable for use as TWCs, here we study the evolution of electronic structure as a function of compositional alteration utilizing hard X-ray photoelectron spectroscopy (HAXPES). Alloy nanoparticles (NPs) are prepared by solid solution synthesis and covered with polyvinylpyrrolidone (PVP) to prevent NPs from agglomeration. The ratio of PVP to the overall metallic content can be estimated as large as 20%, resulting in a PVP protective layer with a thickness of a few nm. This thickness of PVP and its insulating nature make it impossible to utilise laboratory X-ray photoelectron spectroscopy (XPS) tools. In contrast, use of hard X-rays can effectively overcome the above-stated limitation of laboratory XPS instruments and provide reliable data about the electronic states of the metals in the alloy NPs. Information that can be obtained from core level (CL) and valence band (VB) data will help determine the optimal composition of alloys to achieve the most stable and catalytically effective NPs. Note that the Rh_*x*_Cu_*y*_ NPs investigated in this work exhibit comparable catalytic activity (Supplementary Figure SI 2). Experimentally obtained electronic structure data provide essential inputs for theoretical calculation of realistic alloy NP models, including orbital projected DOS. Such models will aid overall understanding and help to classify the phenomena responsible for electronic structure modification during alloying. The ultimate goals are to control and predict the physicochemical properties of new alloy NPs.

## Results and Discussion

### CL HAXPES

**Figure 1:**
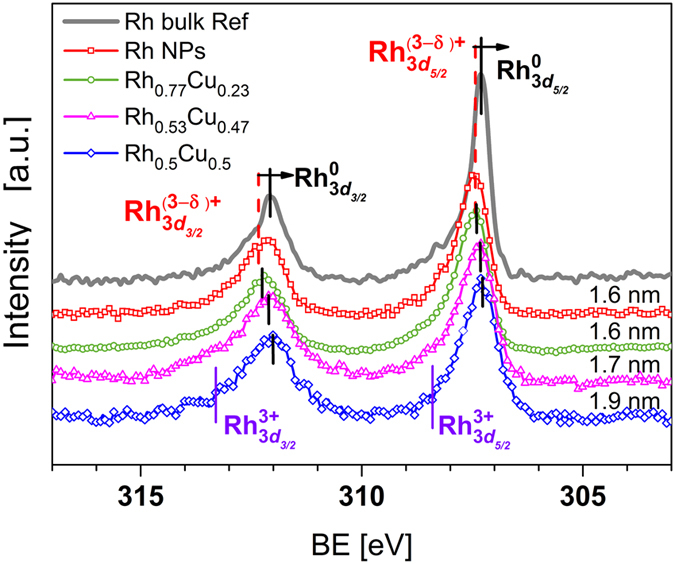
Rh 3*d* CL HAXPES data for reference Rh (solid line), monometallic Rh NPs (red square scatters), Rh_0.77_Cu_0.23_ (green circle scatters), Rh_0.53_Cu_0.47_ (magenta triangle scatters) and Rh_0.50_Cu_0.50_ (blue diamond scatters) alloy NPs. The main peak position of monometallic Rh NPs is shifted towards higher BE compared with that of the reference sample, indicating partial oxidation. With increasing Cu content of the bimetallic alloy NPs, the main peak position recovers to that of Rh^0^, indicating intermetallic charge transfer from Cu to Rh in the alloy NPs. The main peak positions for Rh reference are consistent with the Rh^0^ BE reported elsewhere^23^,^24^,^25^,^26^.

**Figure 2:**
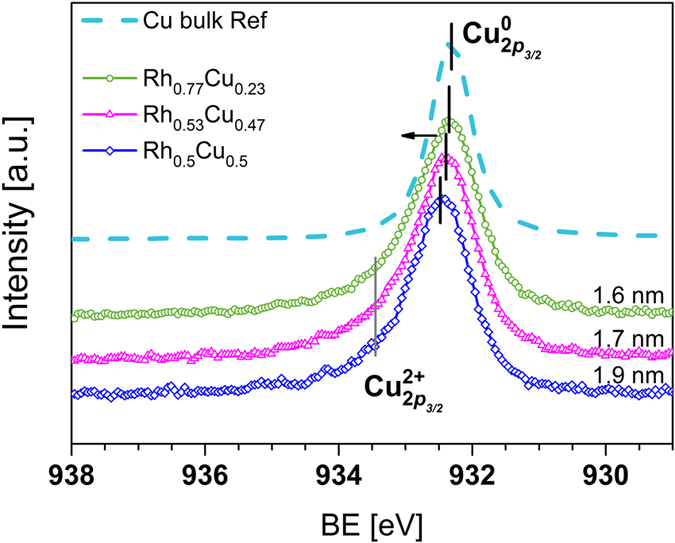
Cu 2*p*_3/2_ CL HAXPES data for reference Cu (dashed line), and Rh_0.77_Cu_0.23_ (green circle scatters), Rh_0.53_Cu_0.47_ (magenta triangle scatters) and Rh_0.50_Cu_0.50_ (blue diamond scatters) alloy NPs. The main peak position of all alloy NPs shifted towards higher BE compared with that of the reference sample, indicating size-dependent partial oxidation of Cu. The main peak positions for the reference are consistent with those reported for metallic Cu^27^,^28^,^29^.

HAXPES measurements were performed to identify changes in the electronic structure of mono- and bimetallic NPs with different compositions formed by solid solution synthesis. Figures 1 and 2 show the Rh 3*d* and Cu 2*p*_3/2_ CL HAXPES spectra, respectively, of investigated samples along with those of reference bulk metals. The binding energies (BEs) of Rh 3*d* and Cu 2*p*_3/2_ for mono- and bimetallic alloy NPs and reference metals are summarised in Table 1. The peak positions and their assignments are consistent with reported data^23^,^24^,^25^,^26^,^27^,^28^,^29^. It should be mentioned that, within the standard deviation of transmission electron microscopy (TEM) results (refer to Supplementary Figure SI 1), the size of NPs discussed here can be regarded as similar. Therefore, the discussion is conducted in terms of evaluation of electronic structure modification with compositional alteration of the alloy excluding size effects.

The change in the electronic structure observed as a result of alloying of Rh and Cu can be understood as concurrent reduction of Rh and oxidation of Cu. Figures 1 and 2 reveal that the CL peaks for Rh 3*d* and Cu 2*p*_3/2_ move in opposite directions, indicating the presence of an intermetallic interaction, which can be viewed as charge transfer from Cu to Rh. This observation is consistent with the Miedema model of metals and alloys, where charge transfer is related to the electronegativity of constituents and typically occurs from atoms of lower to atoms of higher electronegativity^30^. Thus, intuitively for Rh_*x*_Cu_*y*_ alloy, intermetallic charge transfer is expected to occur from Cu to Rh because the electronegativity values for Cu and Rh are 1.90 and 2.28 according to the Pauling scale, respectively^31^. Even though, the Miedema semi-empirical theory can not predict all features of an alloy, it is applicable to the description of investigated samples. The assumption of charge transfer is further supported by the CL HAXPES data presented here. In particular, with decreasing Rh content, the Rh 3*d* BEs of the bimetallic alloy NPs shift to lower energy, which is consistent with the reduction of Rh. This trend is clearly seen in Fig. 1 for the peak positions of Rh 3*d*_5/2_ and Rh 3*d*_3/2_. For the Rh 3*d* peaks, their difference in BE varied from about 0.15 to 0.28 eV as alloy composition altered. With a given experimental resolution (refer to the Methods section), this can be treated as detectable (e.g. worth discussion) but not as a drastic difference. At the same time, the Cu 2*p*_3/2_ BEs of the bimetallic alloy NPs are shifted to higher energies, indicating the partial oxidation of Cu. We should note that the presence of partly oxidised Cu is beneficial for the catalyst; namely, in CO conversion. This also explains the slight improvement of CO conversion data for Rh_*x*_Cu_*y*_ alloy compared with that for monometallic Rh NPs, as can be seen in Figure SI 2(c). Once oxygen atoms are present on the surface of NPs, CO can react with O and desorb as CO_2_. The synthesis conditions of Rh_*x*_Cu_*y*_ NPs and use of PVP, with chemically active oxygen and nitrogen, will tolerate partial oxidation of metals ions. For bimetallic Rh_*x*_Cu_*y*_ alloys, formation of complex Cu oxides inevitably affects the adsorption of ligands on Rh atoms, preventing oxidation of the latter. This trend is most prominent for Rh_*x*_Cu_*y*_ alloy NPs with comparable x:y ratio. In other words, Cu acts as a sacrificial anode to avoid the oxidation of Rh. Because all the NPs are capped with PVP, we assumed that the influence of this protective agent on the electronic structure of the NPs was the same in all cases, which is confirmed by the similar CL N 1*s* signal detected for all the NP samples (refer to Supplementary Figure SI 3). Therefore, we conclude that the changes observed in Rh 3*d* and Cu 2*p*_3/2_ CL HAXPES data originated from changes in the electronic structure of the NPs as a function of compositional alteration (different x:y ratios).

Previously reported size-dependent changes in the catalytic activity of Rh NPs indicate that smaller Rh NPs are more catalytically active than larger ones. We should stress that a difference in size of more than few nm is considered to be important for size-dependent studies. Such size-dependent changes in activity are attributed to changes in the ratio of rhodium surface oxide with a non-integer oxidation state (Rh^(3−*δ*)+^) to metallic Rh with NP size. For smaller NPs, a higher ratio of (Rh^(3−*δ*)+^) oxide leads to increased catalytic activity^32^. For example, Grass *et al*.^24^ proposed a scenario in which the presence of an interface between the metallic core and surface metal oxide layer induces strain in the oxide, making it less stable and thus more reactive.

Formation of surface oxide is clearly seen in the Rh 3*d* HAXPES data acquired for monometallic Rh NPs (Fig. 1). The position of the Rh 3*d*_5/2_ peak for the Rh NPs indicates the coexistence of metallic Rh^0^ and Rh oxide with a non-integer state Rh^(3−*δ*)+^. Formation of complex Rh NPs that contain metallic Rh^0^ and Rh oxide with a non-integer state is favourable because it enhances CO oxidation, whereas a fully oxidised Rh_2_O_3_ surface layer will poison the reaction^33^,^34^,^35^. For this reason, we conclude that the catalytic activity observed for the monometallic Rh NPs, which is comparable to that of bulk Rh, originates from a surface Rh oxide layer with a non-integer oxidation state Rh^(3−*δ*)+^.

**Figure 3:**
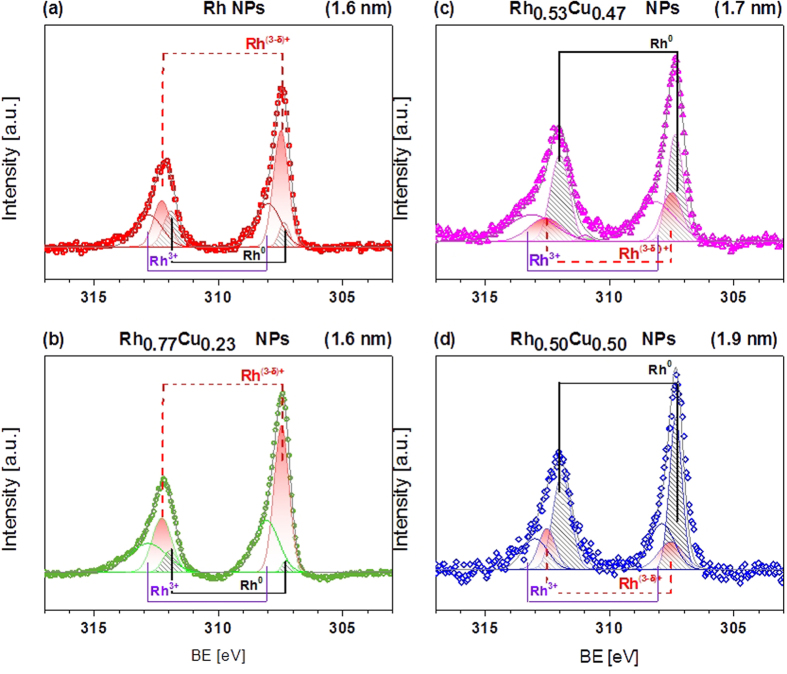
Fitting results of Rh 3*d* CL HAXPES data for (**a**) monometallic Rh NPs, (**b**) Rh-rich Rh_0.77_Cu_0.23_, (**c**) Rh_0.53_Cu_0.47_ and (**d**) Rh_0.50_Cu_0.50_ alloy NPs. Shaded areas are fitting results for metallic Rh^0^ (black) and rhodium oxide with non-integer state Rh^(3−*δ*)+^ (red).

As mentioned above, for bimetallic Rh_*x*_Cu_*y*_ NPs with comparable x:y ratio, changes in electronic structure can be understood as simultaneous reduction of Rh and oxidation of Cu, implying that intermetallic charge transfer occurs from Cu to Rh (see the black solid lines used to mark the positions of Rh 3*d* and Cu 2*p*_3/2_ peaks in Figs 1 and 2). This statement is in agreement with the fitting results of Rh 3*d* CL HAXPES data shown in Fig. 3. The fitting results of CL HAXPES data indicate that the high catalytic activity of Rh-rich NPs (monometallic Rh and Rh_0.77_Cu_0.23_ NPs) originates from their relatively high ratio of reactive Rh^(3−*δ*)+^ oxide to metallic Rh formed during their solid solution synthesis, in agreement with previously reported data^34^. Remarkably, intermetallic interactions are even more important for bimetallic alloy NPs with comparable x:y ratio; e.g., Rh_0.53_Cu_0.47_ and Rh_0.50_Cu_0.50_ NPs. For these samples, the fitting results of Rh 3*d* CL HAXPES data reveal a clear decrease in content of the reactive Rh^(3−*δ*)+^ oxide component (Fig. 3(c,d)). Based on the above reasoning, this should cause a noticeable decrease in the catalytic activity of Rh_0.53_Cu_0.47_ and Rh_0.50_Cu_0.50_ compared with that of monometallic Rh and Rh_0.77_Cu_0.23_ NPs, which is not the case. Although the content of the reactive Rh^(3−*δ*)+^ component decreases, Cu^2+^ simultaneously emerges in Rh_0.50_Cu_0.50_ and Rh_0.53_Cu_0.47_ alloy NPs (refer to the Cu 2*p* fitting results shown in the Supplementary Figure SI 5 (d) and (c), respectively). Previous studies report promotion of NOx reduction with CO of Cu-based catalysts supported on *γ*-Al_2_O_3_^36^,^37^,^38^. For example, Amano *et al*.^36^ stated that the catalytic reduction of NO with CO was promoted by the redox behaviour of Cu^2+^/Cu^1+^; at the same time, catalytic oxidation of CO with O_2_ was promoted by the redox cycle between Cu^2+^ and Cu^0^ species at temperatures above 300 °C. The temperature-dependent catalytic activity data presented in Supplementary Figure SI 2 indicate that for Rh_*x*_Cu_*y*_ alloy NPs conversion of NOx and CO are dominant reactions because they occur at lower temperatures (around 200 °C) than hydrocarbon conversion (around 260 °C). We argue that intermetallic charge transfer helps to compensate for the decreased fraction of catalytically active Rh^(3−*δ*)+^ in Rh_0.53_Cu_0.47_ and Rh_0.50_Cu_0.50_, thus resulting in negligible change in the catalytic activities of these samples compared with that of Rh-rich NPs.

From the discussion above, it becomes evident that Rh_*x*_Cu_*y*_ alloys with different compositions can be roughly divided into three groups: (i) Rh-rich, represented by monometallic Rh and Rh_0.77_Cu_0.23_ NPs, (ii) alloys with comparable x:y ratio like Rh_0.50_Cu_0.50_ and Rh_0.53_Cu_0.47_ NPs and (iii) Cu-rich NPs, which are not considered here because of challenges related with stabilisation of the nanoscale Cu^0^ phase during synthesis. In this regard, the inclusion of an additional samples into our discussion, for example, NPs with x ≥ 0.6, will presumably strengthen our current line of reasoning but will not provide much qualitatively new information because those NPs will fall into category (i); e.g. Rh-rich alloys.

### VB HAXPES

**Figure 4:**
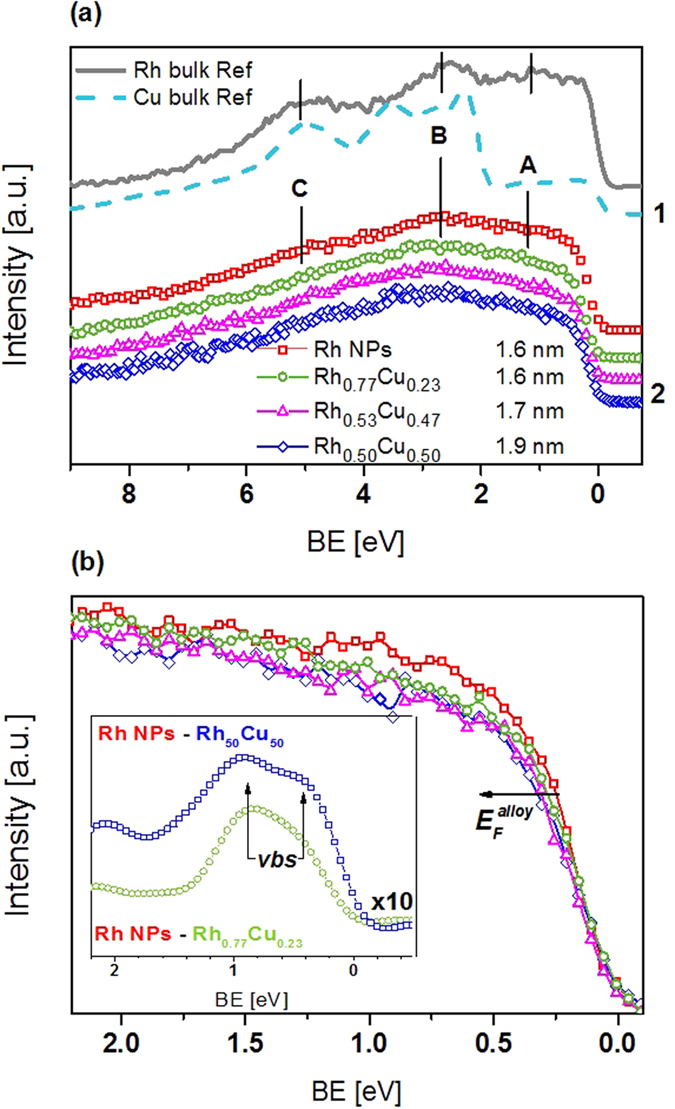
(**a**) VB HAXPES and (**b**) region near Fermi edge of Rh_0.77_Cu_0.23_, Rh_0.53_Cu_0.47_ and Rh_0.50_Cu_0.50_ alloy NPs. Rh and Cu bulk reference are shown in (**a**) section 1. The triplet structure characteristic for metallic Rh is labeled as peaks A-C. The energies of triplet structure are consisted with previously reported experimental data^41^ and theoretical calculations^51^. Inset in (**b**) is the magnified differential spectra in the VB region near the Fermi edge. Maximum of differential spectra is located around 1 eV below Fermi level and associated with formation of Rh 4*d* and/or Rh-Cu virtual bound states (*vbs*) as a result of orbital hybridization caused by alloying. The position of (*vbs*) agreed well with data reported earlier for CuRh alloy^8^.

The comparable CO-oxidising catalytic activity obtained for the samples intuitively implies that there is no drastic change in the electronic structure of the NP alloys as a function of the x:y ratio. This behaviour was confirmed by VB HAXPES measurements, as shown in Fig. 4(a). To allow comparison, all data were collected during the same beam time and the energy range of recorded VB was kept identical. Tougaard background was subtracted and the integrated area was used for signal normalisation. Additionally, section (1) of Fig. 4(a) shows the reference VB spectra of bulk Rh and Cu. Note that even though the VB spectra of the studied NPs lack pronounced fine structure, this is not because of the low experimental resolution of HAXPES measurements, but rather originates from the extremely small size of the NPs. The VB spectrum of bulk Rh acquired under similar experimental conditions supports this statement; it contains a well-resolved triplet structure characteristic of metallic Rh, as indicated by peak A-C in Fig. 4(a). For all NPs, the main contribution to the total DOS in the VB region is from degenerated Rh 4*d* states (cumulative effect of t_2*g*_ and e_*g*_ states^39^), because the photoionization cross-section of Rh 4*d* at 6 keV is approximately 6 times that for Cu 3*d*^40^. The energies of Rh 4*d* triplet structure A-C are consistent with previously reported experimental data as well as theoretical calculations^41^,^42^,^43^,^44^. In addition, reported VB data for larger monometallic Rh NPs (12.2 ± 1.6 nm) prepared in a similar manner qualitatively resemble the VB spectrum of bulk Rh^41^.

For monometallic Rh NPs, triplet structure A-C is resolved, although its respective intensities are less pronounced than those of bulk Rh. The Fermi edge (E_*F*_) of the NPs shifted to a higher energy compared with that of bulk Rh. These differences are attributed to the cumulative effects of (i) extremely small particle size^45^, (ii) narrowing of the d-band as a result of alloying (decreased overlap of the d-orbitals when the neighboring position around a given metal atom is occupied by another component of the alloy) and (iii) the influence of the surface layer (insulating PVP), which cannot be neglected for such small NPs. No VB HAXPES data for alloy NPs with extremely small particle size, such as those in this study, have been reported previously. The VB spectra of alloy NPs displayed in section (2) of Fig. 4(a) are qualitatively similar. Nevertheless, noticeable differences are observed upon comparing the VB HAXPES data for Rh-rich NPs and alloy NPs with comparable Rh:Cu ratio. While all other spectral features are very similar, the VB spectrum of the Rh-rich NPs is more intense in the BE range from 0.25 to 2.2 eV than that of the alloy NPs with comparable Rh:Cu ratio. This region of the VB spectra is worth studying in detail because the electronic structure near E_*F*_ affects catalytic activity. To emphasise observed differences in the VB spectra near E_*F*_, magnified differential spectra are provided in the inset of Fig. 4(b).

For the alloy NPs, the lowering of DOS near E_*F*_ indicates that the alloy NPs are electron deficient relative to the bulk (supported by the CL fit). A possible explanation for this may be the formation of a virtual bound state (*vbs*) associated with Rh 4*d* and/or Rh-Cu bonds as a result of orbital hybridisation caused by alloying. The theory of a virtual bound state was first introduced by Friedel^46^ and Anderson^47^, and is also known as a model that describes the importance of *s-d* exchange (compensating of missing *d* charge with *s* charge) in alloys. Later, Seib and Spicer^48^ proved this theory for CuNi and Ishii *et al*.^8^ applied it to CuRh alloys. The latter researches estimated that the position of the virtual bound state was about 1.25 eV below E_*F*_. This is consistent with data shown in the inset of Fig. 4(b) for Rh_0.50_Cu_0.50_ and Rh_0.77_Cu_0.23_ NPs, refer to the guide line marked as (*vbs*). Orbital hybridisation does not exclude the intermetallic charge transfer from Cu to Rh proposed above. On the contrary, hybridisation of Rh 4*d* and Cu 3*d* orbitals should manifest itself as the formation of bonding and antibonding states near E_*F*_ based on general understanding. However, this is not evident from the direct comparison of VB spectra because no additional features are clearly resolved. We should point out that determination of intermetallic charge transfer in metal alloys based solely on VB data is a difficult task, because even electron density calculations cannot precisely define the region of valence charge associated with each atom. However, comparison of VB and linear combination fitting data based on the reference bulk metals strengthens our argument by taking into account that the observed discrepancy is most pronounced at energies near E_*F*_ (refer to Supplementary Figure SI 7(b) and (d)). Additionally, the E_*F*_ shift for an alloy can be calculated by: 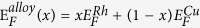, where *x* is the concentration of parent Rh, and 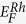 (4.65 eV), 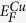 (4.47 eV) and 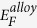, are the Fermi levels of pure Rh, Cu and the alloy, respectively. This equation predicts a progressive shift to higher BE with increasing Cu content, which is consistent with the experimental data presented in Fig. 4(b) (refer to arrow labeled as 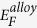). At the same time, the asymmetry of the Cu 2*p*_3/2_ CL peak is small for all NPs, and the asymmetry of Rh 3*d* peaks is smaller for monometallic Rh and Rh-rich alloy NPs than those with a higher Cu content, implying that electron density is lowered at the Cu d-band; in other words, supporting the proposed scenario of intermetallic charge transfer from Cu to Rh. This reasoning is consistent with the findings reported for a Pd_0.50_Cu_0.50_ alloy system^49^,^50^.

**Figure 5:**
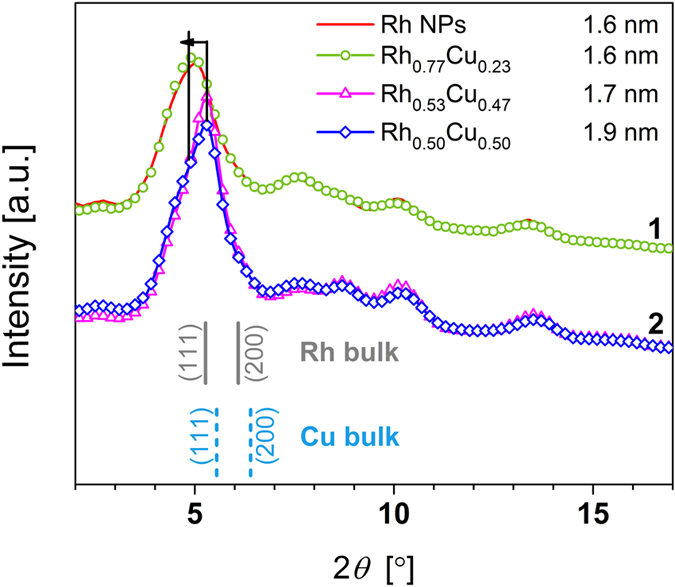
HEXRD pattern of monometallic Rh NPs, Rh_0.77_Cu_0.23_, Rh_0.53_Cu_0.47_ and Rh_0.50_Cu_0.50_ alloy NPs. The position of the first peak of monometallic Rh and Rh-rich NPs is shifted towards lower angle compared with that of alloy NPs with comparable x:y ratio, indicating higher oxide to metal content.

High-energy X-ray diffraction (HEXRD) data, as shown in Fig. 5, confirmed that the investigated samples can be divided into two groups; namely, Rh-rich NPs (section 1 of Fig. 5) and alloy NPs with comparable x:y ratio (section 2 of Fig. 5). Changes observed for the shape and position of the first peak (low 2*θ*) are consistent with conclusions drawn from CL and VB HAXPES data analysis. As described above, the electronic structure of the Rh-rich NPs is dominated by the formation of Rh oxide with a non-integer valence state. Generally, the d spacing of metal oxides is larger than that of the bulk metal. In angular-dependent XRD patterns, this should manifest as a shift of the main peak to lower 2*θ* value. This trend is clearly observed for the Rh-rich alloy NPs (section 1 of Fig. 5). For the alloy NPs with a higher fraction of metallic Rh, shape and position of the first peak exhibit patterns with increased intensity of the Rh 111 peak at higher (2*θ*) values (section 2 of Fig. 5) compared with that for monometallic and Rh-rich NPs.

## Conclusions

In conclusion, the change in electronic structure of extremely small Rh_*x*_Cu_*y*_ alloy NPs with different x:y ratios was investigated by CL and VB HAXPES, confirming that the electronic structure of these NPs is a function of their composition. For monometallic Rh and Rh-rich alloy NPs, formation of Rh surface oxide with a non-integer oxidation state (Rh^(3−*δ*)+^) is the main factor that leads to their high catalytic activity. Conversely, for alloy NPs with comparable x:y ratio (Rh_0.53_Cu_0.47_ and Rh_0.50_Cu_0.50_), the decreased fraction of catalytically active Rh^(3−*δ*)+^ oxide is compensated for by charge transfer from Cu to Rh, resulting in negligible change in their catalytic activities compared with those of Rh-rich and monometallic Rh NPs.

## Methods

### Synthesis of Rh_*x*_Cu_*y*_ alloy NPs

Rh_*x*_Cu_*y*_ alloy NPs with a narrow size distribution and diameter of about 2 nm were prepared by chemical reduction using rhodium(III) acetate Rh(AcO)_3_ and copper(II) chloride CuCl_2_ as metal precursors, and poly(N-vinyl-2-pyrrolidone) (PVP) as a protective agent. The strong base potassium tert-butoxide (CH_3_)_3_COK was added to control the pH of the ethylene glycol solution, which was used as a reducing agent and buffer medium. The fabrication details of the alloy NPs can be found elsewhere^51^.

### Characterisation and catalytic activity of Rh_*x*_Cu_*y*_ alloy NPs

TEM analyses revealed that within the standard deviation, the size of Rh_*x*_Cu_*y*_ alloy NPs was independent of the Cu and Rh content and remained the same (at about 2 nm) for all samples (refer to Supplementary Figure SI 1). The x:y ratio in the prepared alloy NPs was determined by energy-dispersive X-ray elemental analysis. To investigate their catalytic activity, Rh_*x*_Cu_*y*_ NPs supported on *γ*-Al_2_O_3_ catalysts were prepared by wet impregnation. Details of the wet impregnation process can be found elsewhere^51^. The Rh_*x*_Cu_*y*_ NPs with different compositions exhibited comparable catalytic activity (Supplementary Figure SI 2).

### Hard X-ray photoelectron spectroscopy (HAXPES)

HAXPES CL and VB spectra for samples were recorded at the National Institute for Materials Science (NIMS) contract undulator beamline BL15XU at SPring-8, Japan^52^,^53^. A linearly polarised X-ray beam with an incident photon energy of 5.95 keV was used for HAXPES measurements. Use of hard X-rays enabled bulk-sensitive measurements to be performed. A high-resolution hemispherical analyser (VG Scienta R4000) was used to collect spectra. All data were recorded at room temperature with the take-off angle of photoelectrons with respect to the sample holder surface set to 88°. For an incident photon energy of 5.95 keV, the total energy resolution estimated using an Au reference sample was 240 meV. A combination of Tougaard and linear backgrounds was subtracted from the spectra and BEs were calibrated with respect to the Fermi edge of the Au reference. The effective information depth was approximately 17–20 nm, which was estimated using the equation proposed by Tanuma, Powell, and Penn (TPP-2M)^54^. This depth is an order of magnitude greater than the largest Rh_*x*_Cu_*y*_ NPs, indicating that the data provided a reliable account of the electronic structure of the NPs.

### High energy X-ray diffraction (HEXRD) experiments

HEXRD data were obtained by two-axis diffractometer installed at the beamline BL04B2 at SPring-8, Japan. The incident synchrotron X-ray of 61.37 keV was generated using an Si(111) monochromator. The accessible Q range was found to be 0.2 to 25 Å^−1^ by measuring the scattering angle range from 0.3 to 48.9° at intervals of 0.1° with an exposure time of 20 s using three CdTe detectors. The HEXRD measurements for all NP samples were performed in symmetric transmission geometry at room temperature.

## Additional Information

**How to cite this article**: Palina, N. *et al*. Electronic Structure Evolution with Composition Alteration of Rh_*x*_Cu_*y*_ Alloy Nanoparticles. *Sci. Rep.* **7**, 41264; doi: 10.1038/srep41264 (2017).

**Publisher's note:** Springer Nature remains neutral with regard to jurisdictional claims in published maps and institutional affiliations.

## Supplementary Material

Supplementary Information

## Figures and Tables

**Table 1:** Summary of binding energies (BE) of Rh 3*d* and Cu 2*p*_3/2_ representing position of the peak with dominant spectral weight text.

Sample	Rh^0^ 3*d*_5/2_	Rh^(3−*δ*)+^ 3*d*_5/2_	Rh^0^ 3*d*_3/2_	Rh^(3−*δ*)+^ 3*d*_3/2_	Cu^0^ 2*p*_3/2_	Cu^2+^ 2*p*_3/2_
Rh bulk	307.3	—	312.00	—	—	—
Rh NPs	—	307.45	—	312.26	—	—
Rh_0.77_Cu_0.23_	—	307.43	—	312.25	932.34	933.40
Rh_0.53_Cu_0.47_	307.32	—	311.97	—	932.37	933.38
Rh_0.50_Cu_0.50_	307.30	—	311.90	—	932.45	933.40
Cu bulk	—	—	—	—	932.33	933.51
