# Supplementary material for: Electronic Structure Evolution with Composition Alteration of RhxCuy Alloy Nanoparticles
Source: Sci Rep. 2017 Jan 25;7:41264. doi: 10.1038/srep41264 (PMC5264589; doi:10.1038/srep41264)
Supplement: Supplementary Information [file srep41264-s1.pdf]

SUPPLEMENTARY INFORMATION
Electronic Structure Evolution with Composition Alteration of Rh_xCu_y Alloy Nanoparticles

Natalia Palina[1,*], Osami Sakata[1,2,3,*], L. S. R. Kumara[1], Chulho Song[1], Katsutoshi Sato[4,5], Katsutoshi Nagaoka[5], Tokutaro Komatsu[6], Hirokazu Kobayashi[7], Kohei Kusada[7], and Hiroshi Kitagawa[7,8,9]

[1]Synchrotron X-ray Station at SPring-8, Research Network and Facility Services Division, National Institute for Materials Science (NIMS), 1-1-1 Kouto, Sayo-cho, Sayo-gun, Hyogo 679-5148, Japan
[2]Synchrotron X-ray Group, Research Center for Advanced Measurement and Characterization, NIMS, 1-1-1 Kouto, Sayo-cho, Sayo-gun, Hyogo 679-5148, Japan
[3]Department of Materials Science and Engineering, School of Materials and Chemical Technology, Tokyo Institute of Technology, 4259-J3-16, Nagatsuta, Midori, Yokohama 226-8502, Japan
[4]Elements Strategy Initiative for Catalysts and Batteries, Kyoto University, 1-30 Goryo-Ohara, Nishikyo-ku, Kyoto 615-8245, Japan
[5]Department of Applied Chemistry, Faculty of Engineering, Oita University, 700 Dannoharu, Oita 870-1192, Japan
[6]School of Medicine, Nihon University, 30-1, Oyaguchi Kami-cho, Itabashi-ku, Tokyo 173-8610 Japan
[7]Division of Chemistry, Graduate School of Science, Kyoto University, Kitashirakawa Oiwake-cho, Sakyo-ku, Kyoto 606-8502, Japan
[8]Institute for Integrated Cell-Material Sciences (iCeMS), Kyoto University, Yoshida, Sakyo-ku, Kyoto 606-8501, Japan
[9]INAMORI Frontier Research Center, Kyushu University, 744 Motooka, Nishi-ku, Fukuoka 819-0395, Japan
[*]MUELLER.Natalia@nims.go.jp
[*]SAKATA.Osami@nims.go.jp

ABSTRACT

The change in electronic structure of extremely small Rh_xCu_y alloy nanoparticles (NPs) with composition variation was investigated by core-level (CL) and valence-band (VB) hard X-ray photoelectron spectroscopy. A combination of CL and VB spectra analyses confirmed that intermetallic charge transfer occurs between Rh and Cu. This is an important compensation mechanism that helps to explain the relationship between catalytic activity and composition variation of the Rh_xCu_y alloy NPs. For monometallic Rh and Rh-rich alloy ($Rh_{0.77}Cu_{0.23}$) NPs, the formation of Rh surface oxide with a non-integer oxidation state ($Rh^{(3-\delta)+}$) resulted in higher catalytic activity. Whereas, for alloy NPs with comparable Rh:Cu ratio ($Rh_{0.53}Cu_{0.47}$ and $Rh_{0.50}Cu_{0.50}$), the decreased fraction of catalytically active $Rh^{(3-\delta)+}$ oxide is compensated by charge transfer from Cu to Rh. As a result, ensuring negligible change in the catalytic activities of the NPs with comparable Rh:Cu ratio to those of Rh-rich and monometallic Rh NPs.

Initial characterization

Transmission Electron Microscopy

Transmission-electron microscopy (TEM) images were recorded by using Hitachi HT7700 with the acceleration voltage of 100 kV. TEM images and respective size distribution histogram are shown in Figure SI 1. On the basis of TEM results and their standard deviation the size of NPs studied in this work can be treated as almost the same. Therefore, size effects are not considered in the discussion related to the electronic structure modification with compositional alteration.

Three-way catalytic activity measurement

The synthesized Rh_xCu_y alloy NPs were supported on γ-Al_2O_3 catalysts by wet impregnation. The amount of NPS was 1 wt % of γ-Al_2O_3. Temperature dependent three-way catalytic activity was measured on a gas mixture of CO: 5046 ppm, O_2: 4920 ppm, H_2: 1758 ppm, CO_2: 12.45 %, C_3H_6: 467 ppm, NO: 1161 ppm and He at a flow rate of 200 ml min^{-1} with a sample

amount of 0.2 g (SV=60,000 mL/gh), where SV is space velocity. Temperature dependent catalytic activity data are shown in Figure SI 2 for (a) NO_x , (b) CO and (c) hydrocarbon conversions to less harmful N_2, CO_2 and water respectively. Please note that catalytic activity data for $Rh_{0.53}Cu_{0.47}$ are very similar to the data obtained for $Rh_{0.50}Cu_{0.50}$ NPs and was omitted in order to avoid visual overload in Figure SI 2. Additionally, the NPs with $x \geqslant 0.6$ are intuitively fall into category of Rh-rich alloys and hence, the inclusion of these samples will strengthen discussion but hardly will provide qualitatively new information, hence not shown here.

Core level and valence band HAXPES

Influence of polyvinylpyrrolidone (PVP)

Alloy NPs were prepared by solid-solution synthesis and covered with an polyvinylpyrrolidone (PVP) to prevent NPs from agglomeration. The ratio of PVP to the overall metallic content was estimated as large as 20 wt %, resulting in the thickness of protective agent about few nm. For NPs of 2 nm size this is a substantial fraction. The overall effect: (i) chemical reactivity and (ii) its insulating nature, of PVP should not be underestimated. Figure SI 3 and Figure SI 4 show N 1*s* and C 1*s* core-level (CL) spectra for NPs and including bulk references (C 1*s* CL spectra). Acquired CL HAXPES data showed similar trend for all samples, reassuring that influence of the protective agent on electronic structure of NPs is the same. Therefore, all differences which are discussed in the manuscript, are originate from the change in electronic structure as a function of compositional alteration.

Cu 2*p* CL fit results

Fit results of Cu 2*p* CL HAXPES data clearly indicate an oxidation of Cu (see Figure SI 5 shadowed area indicative for Cu^{2+}) for alloy NPs with comparable Rh:Cu ratio (2nd group). This finding supports presence of charge transfer mechanism from Cu to Rh as suggested based on the fit results of Rh 3*d* CL HAXPES data shown in the main manuscript. Whereas, Rh-rich alloy NPs exhibit only partial oxidation of Cu, refer to Figure SI 5 (b).

Valence band linear combination fit results

Linear combination fit (LCF) results by fitting each sample spectrum with weighted mixtures (accounting for photo-ionization cross-section) of the bulk Rh and Cu references are shown in Figure SI 6. Our data support presence of: (i) partial oxidation of Rh for monometallic and Rh-rich alloy NPs (1st group) and charge transfer mechanism from Cu to Rh for alloy NPs with comparable Rh:Cu ratio (2nd group). Additionally, LCF analysis failed to reproduce experimental VB spectra, indicating a chemical mixture of Rh and Cu on the atomic level during synthesis. In the energy range near E_F the mismatch between experimental and LCF spectra are most pronounced for alloy NP's with comparable Rh:Cu ratio, supporting intermetallic charge transfer mechanism.

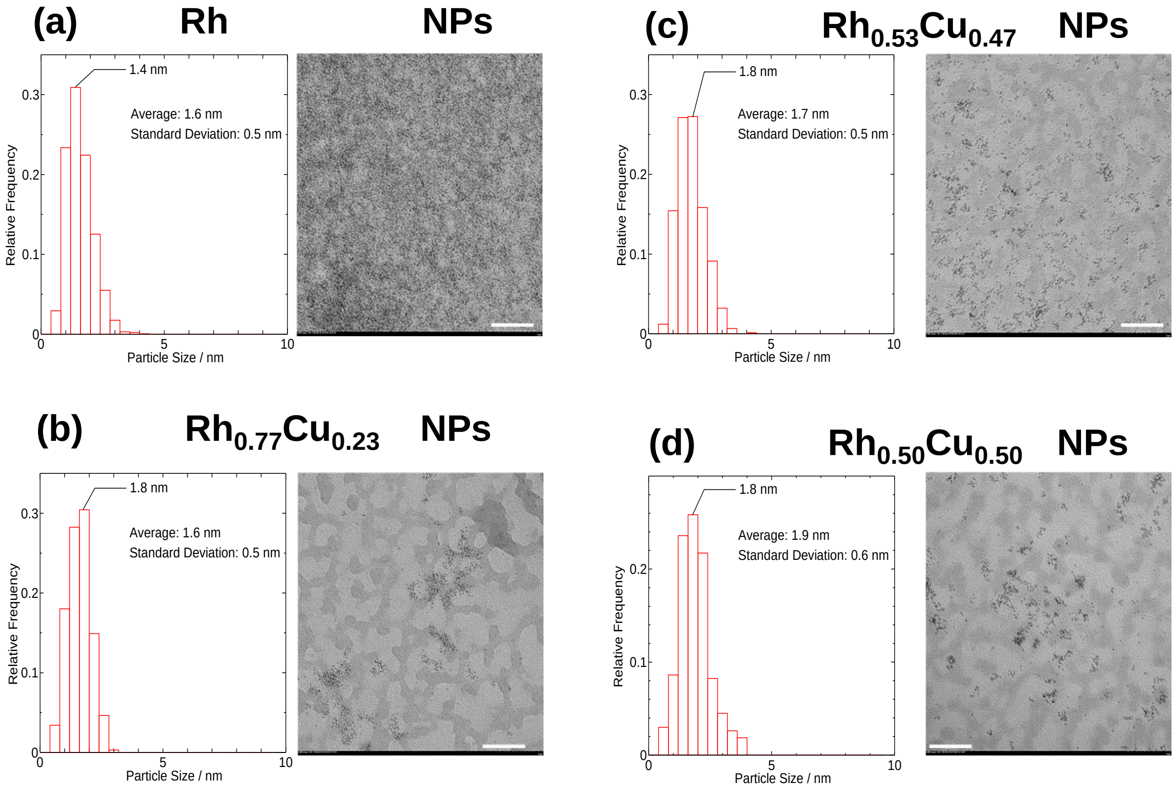

Figure SI 1. TEM images of (a) Rh NPs, (b) $Rh_{0.77}Cu_{0.23}$, (c) $Rh_{0.53}Cu_{0.47}$ and (d) $Rh_{0.50}Cu_{0.50}$ alloy NPs. TEM images indicated that for monometallic Rh NPs the mean size was 1.6±0.5 nm and of the alloy NPs was about 1.6±0.5 nm, 1.7±0.5 nm and 1.9±0.6 nm for, $Rh_{0.77}Cu_{0.23}$, $Rh_{0.53}Cu_{0.47}$ and $Rh_{0.50}Cu_{0.50}$ respectively.

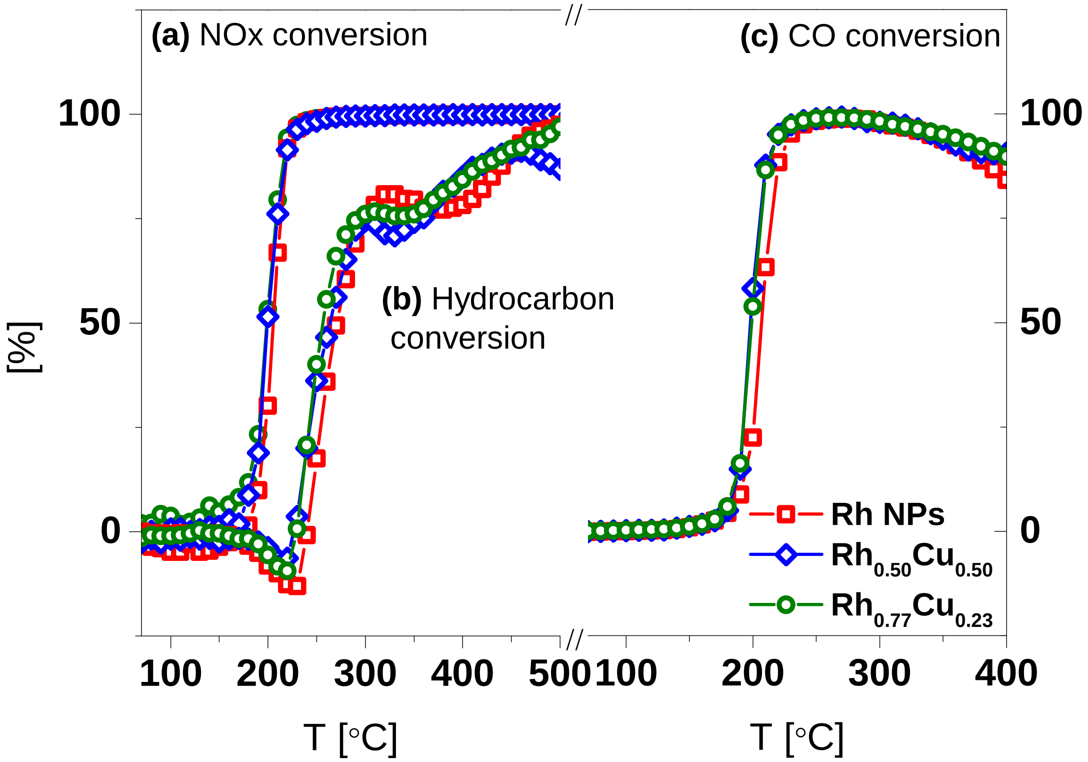

Figure SI 2. Temperature dependent catalytic activity data for (a) NO_x conversion, (b) hydrocarbon conversion and (c) CO conversion for monometallic Rh NPs (red square scatters), $Rh_{0.50}Cu_{0.50}$ (blue diamond scatters) and $Rh_{0.77}Cu_{0.23}$ (green circle scatters) alloy NPs.

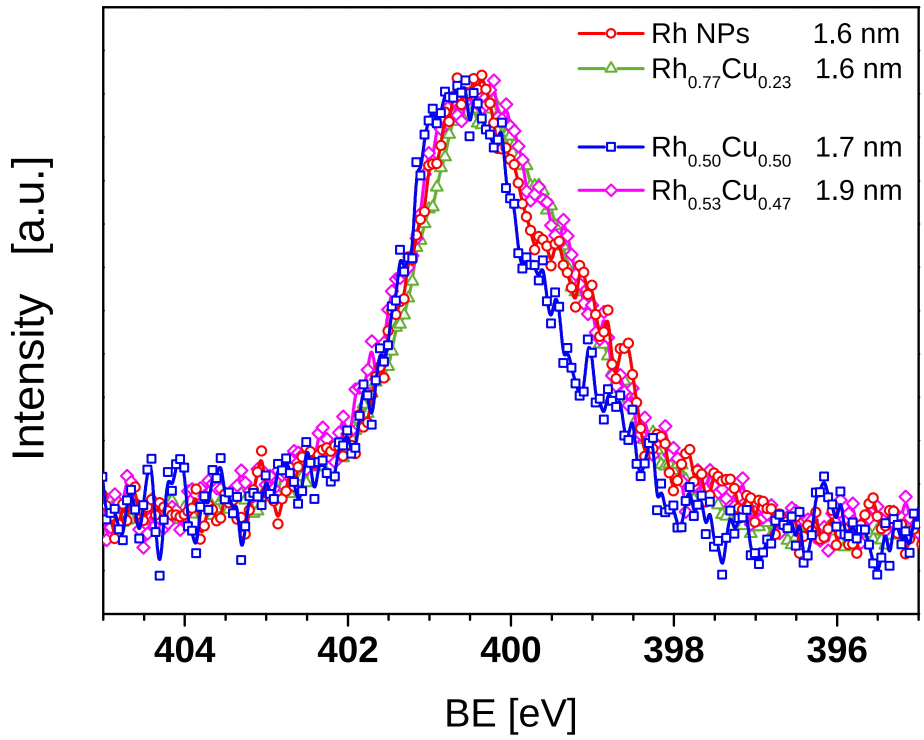

Figure SI 3. N 1*s* CL spectra for monometallic Rh NPs (red square scatters), $Rh_{0.77}Cu_{0.23}$ (green circle scatters), $Rh_{0.53}Cu_{0.47}$ (magenta triangle scatters) and $Rh_{0.50}Cu_{0.50}$ (blue diamond scatters) alloy NPs. Similarities observed for the shape and energy position of CL data supports statement of the main manuscript stating that influence of the protective agent on NPs electronic structure is the same.

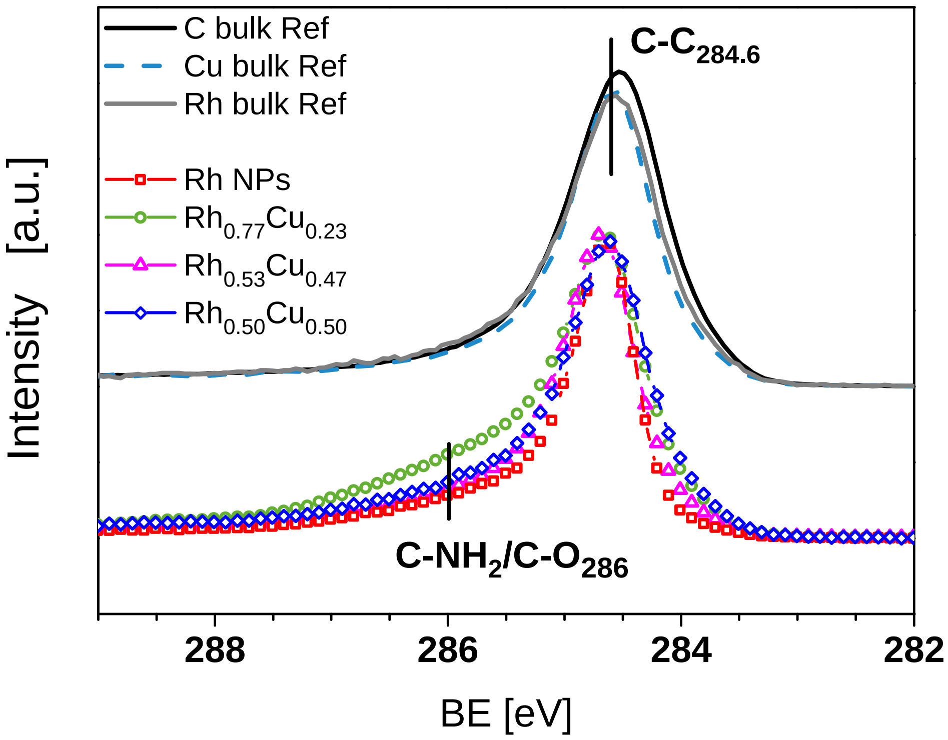

Figure SI 4. C 1*s* CL HAXPES data for monometallic Rh NPs (red square scatters), $Rh_{0.77}Cu_{0.23}$ (green circle scatters), $Rh_{0.53}Cu_{0.47}$ (magenta triangle scatters) and $Rh_{0.50}Cu_{0.50}$ (blue diamond scatters) alloy NPs. CL showing similar trend for all samples, reassuring that possible carbon contamination is identical and originated from PVP.

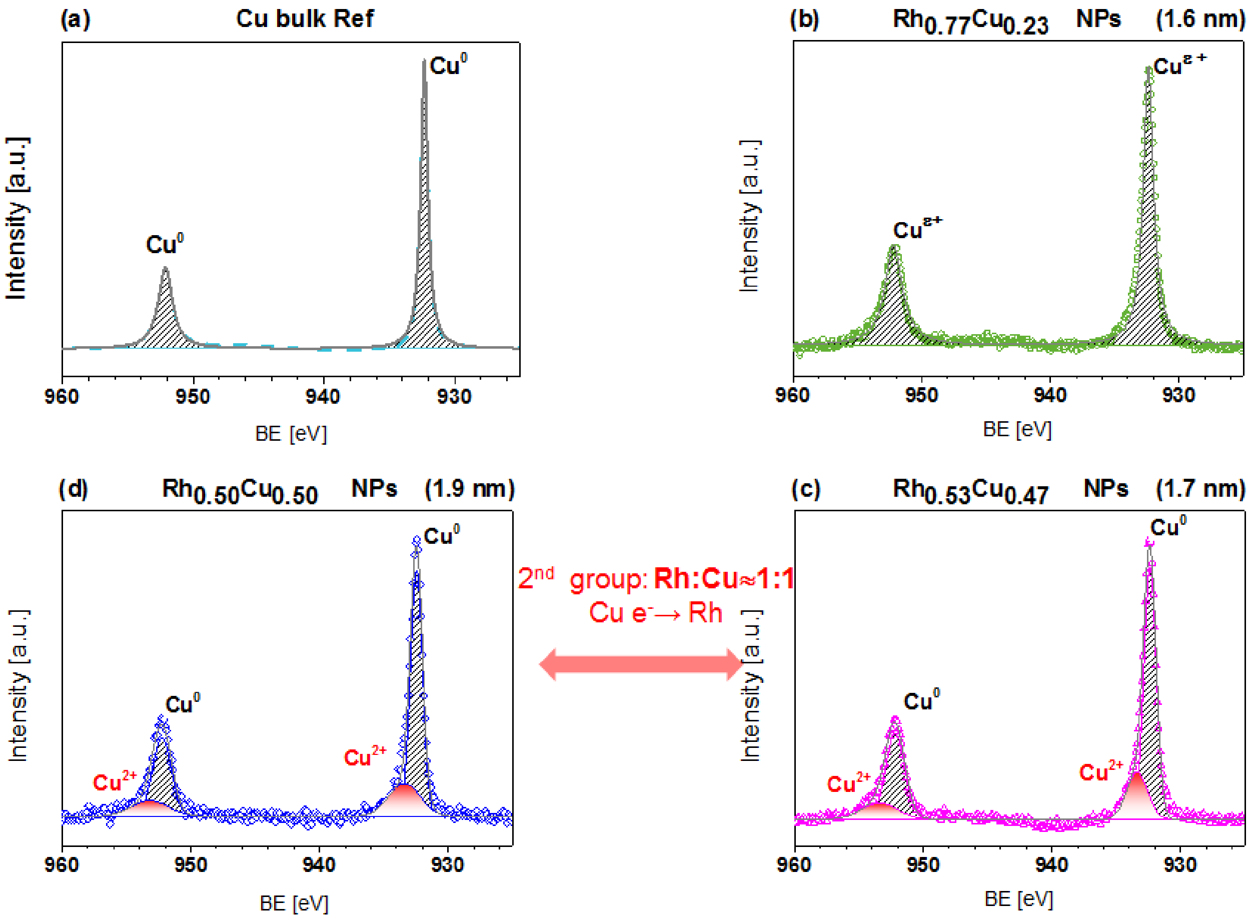

Figure SI 5. Fit results of Cu *2p* CL HAXPES data for (a) Cu bulk reference (dashed line), (b) $Rh_{0.77}Cu_{0.23}$ (green circle scatters), (c) $Rh_{0.53}Cu_{0.47}$ (magenta triangle scatters) and (d) $Rh_{0.50}Cu_{0.50}$ (blue diamond scatters) alloy NPs. CL HAXPES data clearly indicate Cu oxidation (for alloy NPs forming 2nd group), supporting presence of charge transfer mechanism from Cu to Rh.

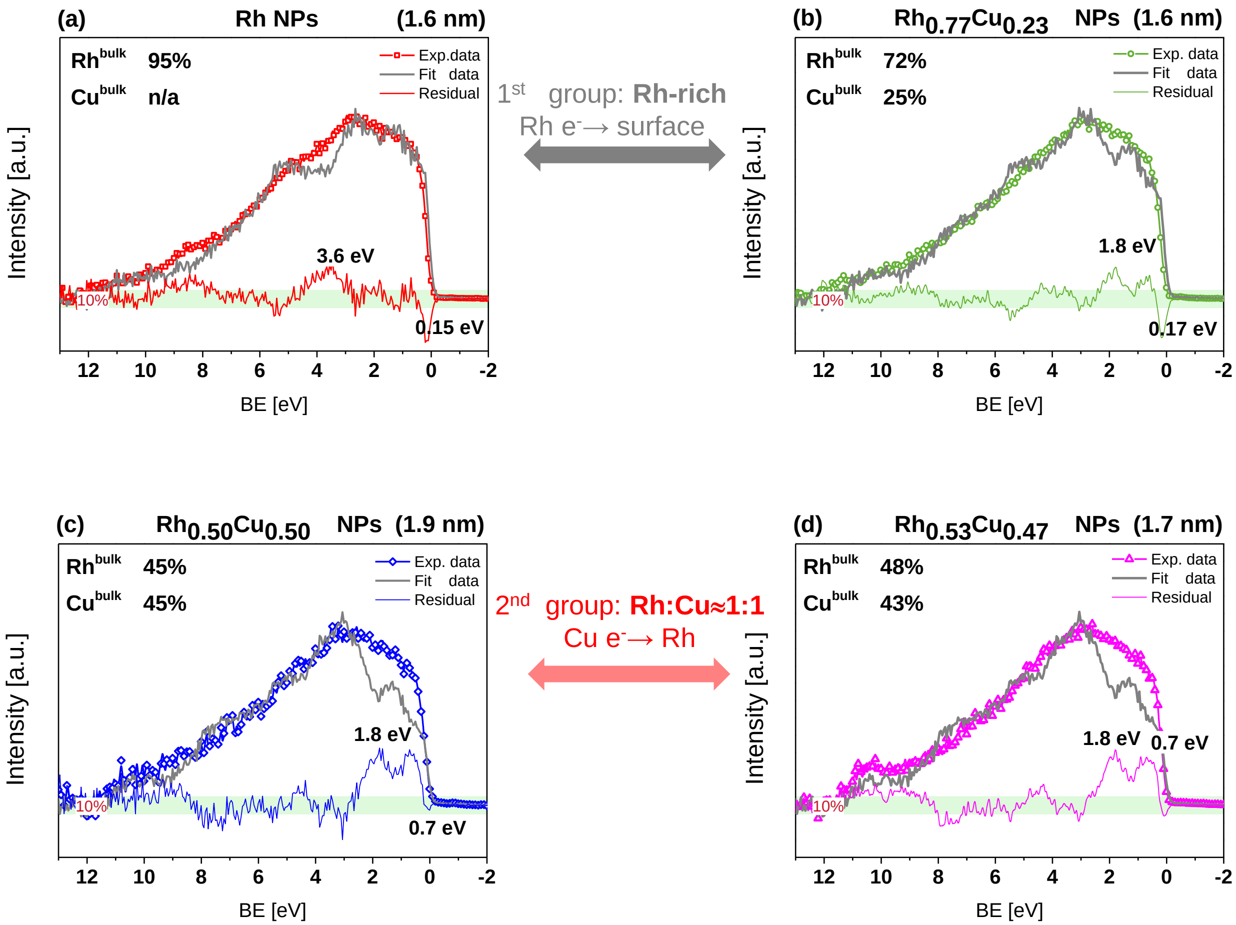

Figure SI 6. VB fit results supporting presence of: (i) partial oxidation of Rh for monometallic and Rh-rich alloy NPs (1st group) and charge transfer mechanism from Cu to Rh for alloy NPs with comparable Rh:Cu ratio (2nd group). Linear combination fit (LCF) analysis based on the bulk reference compounds failed to reproduce experimental VB spectra, indicating a chemical mixture of Rh and Cu on the atomic level during synthesis. In the energy range near E_F the mismatch between experimental and LCF spectra are most pronounced for alloy NP's with comparable Rh:Cu ratio, supporting intermetallic charge transfer mechanism.
